# Interactions between Serum Vitamin D Levels and Vitamin D Receptor Gene Fok*I* Polymorphisms for Renal Function in Patients with Type 2 Diabetes

**DOI:** 10.1371/journal.pone.0051171

**Published:** 2012-12-04

**Authors:** Keitaro Yokoyama, Akio Nakashima, Mitsuyoshi Urashima, Hiroaki Suga, Takeshi Mimura, Yasuo Kimura, Yasushi Kanazawa, Tamotsu Yokota, Masaya Sakamoto, Sho Ishizawa, Rimei Nishimura, Hideaki Kurata, Yudo Tanno, Katsuyoshi Tojo, Shigeru Kageyama, Ichiro Ohkido, Kazunori Utsunomiya, Tatsuo Hosoya

**Affiliations:** 1 Division of Kidney, Hypertension, Department of Internal Medicine, Jikei University School of Medicine, Tokyo, Japan; 2 Division of Molecular Epidemiology, Jikei University School of Medicine, Tokyo, Japan; 3 Shin Kashiwa Clinic, Chiba, Japan; 4 Division of Diabetes, Metabolism and Endocrinology, Department of Internal Medicine, Jikei University School of Medicine, Tokyo, Japan; 5 Division of Clinical Pharmacology and Therapeutics, Jikei University School of Medicine, Tokyo, Japan; University of Tokushima, Japan

## Abstract

**Background:**

We aimed to examine associations among serum 25-hydroxyvitamin D (25OHD) levels, 1,25-dihyroxyvitamin D (1,25OHD) levels, vitamin D receptor (VDR) polymorphisms, and renal function based on estimated glomerular filtration rate (eGFR) in patients with type 2 diabetes.

**Methods:**

In a cross-sectional study of 410 patients, chronic kidney disease (CKD) stage assessed by eGFR was compared with 25OHD, 1,25OHD, and VDR Fok*I* (rs10735810) polymorphisms by an ordered logistic regression model adjusted for the following confounders: disease duration, calendar month, use of angiotensin converting enzyme inhibitors/angiotensin receptor blockers or statins, and serum calcium, phosphate, and intact parathyroid hormone levels.

**Results:**

1,25OHD levels, rather than 25OHD levels, showed seasonal oscillations; peak levels were seen from May to October and the lowest levels were seen from December to February. These findings were evident in patients with CKD stage 3∼5 but not stage 1∼2. eGFR was in direct proportion to both 25OHD and 1,25OHD levels (*P*<0.0001), but it had stronger linearity with 1,25OHD (r = 0.73) than 25OHD (r = 0.22) levels. Using multivariate analysis, 1,25OHD levels (*P*<0.001), but not 25OHD levels, were negatively associated with CKD stage. Although Fok*I* polymorphisms by themselves showed no significant associations with CKD stage, a significant interaction between 1,25OHD and Fok*I*TT was observed (*P* = 0.008). The positive association between 1,25OHD and eGFR was steeper in Fok*I*CT and CC polymorphisms (r = 0.74) than Fok*I*TT polymorphisms (r = 0.65).

**Conclusions:**

These results suggest that higher 1,25OHD levels may be associated with better CKD stages in patients with type 2 diabetes and that this association was modified by Fok*I* polymorphisms.

## Introduction

Worldwide, the number of people with diabetes is projected to rise from 171 million in 2000 to 366 million in 2030 [1]. Chronic kidney disease (CKD) is one of the major complications of type 2 diabetes. CKD in patients with diabetes has become one of the major causes of end-stage renal disease (ESRD) requiring dialysis and transplantation. Therefore, avoiding the development of ESRD in patients with diabetes is important.

25-hydroxyvitamin D (25OHD) is the primary circulating form of vitamin D and is converted into 1,25-dihyroxyvitamin D (1,25OHD) through 1alpha-hydroxyvitamin D (1aOHase); this conversion takes place primarily in the kidneys. As a result, 1,25OHD levels can be decreased in patients with renal dysfunction. Low levels of 1,25OHD increase renal renin production, thus activating the renin-angiotensin-aldosterone system (RAAS), reduce renal expression of klotho, increase fibroblast growth factor-23 levels, and consequently suppress 1aOHase, further lowering 1,25OHD levels, all of which are associated with progression of renal damage [2]. Theoretically, this vicious cycle may be blocked by inhibitors of the RAAS cascade and/or replacement of 1,25OHD. In fact, a meta-analysis demonstrated that angiotensin converting enzyme inhibitors (ACEI) and angiotensin II receptor blockers (ARB) prevented renal morbidity in patients with type 2 diabetes [3]. Moreover, injectable 1,25OHD significantly improved survival of patients on chronic hemodialysis [4]. Paricalcitol, a form of 1,25OHD, was shown to improve albuminuria in pre-dialysis diabetic patients who were receiving ACEI/ARB therapy [5]. On the other hand, serum 25OHD levels are an independent inverse predictor of disease progression and death in patients with stage 2∼5 CKD [6].

Furthermore, Fok*I* polymorphisms in the vitamin D receptor (VDR) gene differ between patients with diabetic nephropathy and healthy subjects [7]. Compared with the Fok*I* TT genotype, Fok*I* CC had 1.7-fold greater function of vitamin D-dependent transcriptional activation of a reporter construct under the control of a vitamin D response element in transfected HeLa cells [8]. Similarly, a 50% effective dose of 1,25-(OH)2D3 in the FokI C/C genotype was significantly lower than in the FokI CT genotype [9]. By switching from ATG (Fok*I* T) to ACG (Fok*I* C) polymorphism, the first potential start site moves to the 3′ direction, resulting in proteins that are three amino acids shorter and more functional [10] in terms of its transactivation capacity as a transcription factor [11].

Taken together, this evidence suggests that 25OHD, 1,25OHD, and VDR may play a role in exacerbation of diabetic nephropathy, at least in part. However, there are no reports studying these three factors together. Therefore, we conducted a cross-sectional study of patients with type 2 diabetes to elucidate 1) which is a stronger factor in the association with eGFR levels and CKD stages, 25OHD or 1,25OHD, and 2) if there is any interaction between 25OHD/1,25OHD and VDR polymorphisms, in association with CKD stages, after adjusting for other confounders.

## Methods

### Study design

This cross-sectional study was carried out as collaboration among the Division of Kidney, Hypertension; Division of Diabetes, Metabolism and Endocrinology; and Division of Molecular Epidemiology, Jikei University School of Medicine. The study protocol was reviewed and approved by the ethics committee of the Jikei Institutional Review Board, Jikei University School of Medicine, as well as the clinical study committee of the Jikei University hospital and Shin-Kashiwa clinic.

### Study population, eligibility, and consent

Patients aged 20 to 80 years old with type 2 diabetes, diagnosed by doctors based on Japanese diagnostic criteria [12] at Division of Diabetes, Metabolism and Endocrinology and Division of Kidney, Hypertension, were eligible and asked to participate in the study by the doctor in charge of the outpatients' clinics. Patients with hyperparathyroidism, mild liver damage, use of active vitamin D, ACEI/ARB or statins, and treated with dialysis, which may affect vitamin D metabolism, were included. The accrual period was from April 2011 to March 2012. All patients provided written informed consent.

### Clinical evaluation

Disease duration (years) was defined as the period of time between diagnosis of diabetes and clinical evaluation for entry into the study. Age, gender, height, weight, and blood pressure as well as laboratory data of peripheral blood calcium (Ca) (normal range: 8.5∼10.4 mg/dL), phosphate (P) (normal range: 2.5∼4.5 mg/dL), and intact parathyroid hormone (iPTH; normal range: 10∼65 pg/mL) levels were recorded. eGFR was calculated according to the following Japanese standard formula based on insulin clearance: 194× creatinine^−1.094^× age^−0.287^, if female, ×0.739 [13]. CKD stages were defined based on eGFR levels as follows: Stage 1 CKD, eGFR ≥90 ml/min/1.73 m^2^; Stage 2 CKD, eGFR ≥60 to <90 ml/min/1.73 m^2^; Stage 3 CKD, eGFR ≥30 to <60 ml/min/1.73 m^2^; Stage 4 CKD, eGFR ≥15 to <30 ml/min/1.73 m^2^; and Stage 5 CKD, eGFR <15 ml/min/1.73 m^2^.

### Samples and 25OHD/1,25OHD measurements

Serum levels of 25OHD (ng/mL) and 1,25OHD (pg/mL) were measured at SRL Inc. (Hachioji, Tokyo, Japan) as described previously [14].

### Polymorphisms of the VDR/GC genes

We used polymerase chain reaction and direct sequencing to analyze VDR polymorphisms according to the Fok*I* single nucleotide polymorphisms as described previously [15].

### Statistical analysis

Associations between CKD stage and patients' characteristics were evaluated using single-ordered logistic regression models and chi-square test. Associations between CKD stage and vitamin D-related factors as well as other possible confounders were evaluated using a multiple-ordered logistic regression model. Hardy-Weinberg equilibrium was assessed by the chi-square test. To identify any interaction between 25OHD/1,25OHD and Fok*I* polymorphisms, we made a new variable by multiplying the 25OHD/1,25OHD levels and the Fok*I*CC/Fok*I*CT/Fok*I*TT polymorphisms. Two-sided *P* values <0.05 were considered statistically significant. All statistical analyses were performed using STATA 12.0 (STATA Corp., College Station, TX).

## Results

### Patients' characteristics

A total of 410 patients agreed to participate in this study. Baseline characteristics of the study population divided by CKD stage are shown in Table 1. Patients with earlier CKD stages had shorter disease duration, were younger except for those with stage 5 disease, and used less ACEI/ARB and statin therapy than patients with advanced stages except for stage 5, and Ca levels were lower in patients with stage 4 and 5 disease. On the other hand, P levels were higher in patients with stage 5 disease. iPTH levels were higher in patients with stage 4 and stage 5 disease. Gender, body mass index, blood glucose, and HbA1c levels were not significantly different among the CKD stages. Active vitamin D was used only for patients with CKD stage 5. However, within the CKD stage 5 patients, 1,25OHD levels were not different between active vitamin D users (14.0±5.0 pg/mL, n = 52) and non-users (12.3±5.8 pg/mL, n = 49).

**Table 1:** Patients' characteristics of the study population and association with eGFR stage * ^1^.

CKD stage (n)	Stage 1 (47)	Stage 2 (162)	Stage 3 (80)	Stage 4 (20)	Stage 5 (101)	P-value
Disease duration (years) median: IQR* ^2^	5:2∼9	10: 5∼17	11: 8∼22	11: 6∼20	16: 10∼23	<0.001* ^3^
Age (years old) median: IQR	55:42∼63	62: 57∼70	70: 60∼76	70: 62∼74	65: 60∼72	<0.001* ^3^
Gender (M/F)	30/17	111/51	63/17	15/5	71/30	0.39* ^4^
Body mass index (kg/m^2^) median: IQR	24:22∼28	24: 22∼27	25: 22∼27	23: 22∼26	23: 20∼26	0.15* ^3^
Usage of ACEI/ARB* ^5^ n (%)	11 (24%)	70 (43%)	52 (65%)	19 (95%)	61 (60%)	<0.001* ^4^
Usage of statin n (%)	16 (35%)	69 (43%)	44 (55%)	11 (55%)	13 (13%)	<0.001* ^4^
Ca (mg/dL) median: IQR	9.2:9.1∼9.4	9.4:9.2∼9.6	9.3:9.0∼9.6	9.0:8.7∼9.2	8.5:8.1∼9.0	<0.001* ^3^
P (mg/dL) median: IQR	3.6:3.2∼4.0	3.4:3.1∼3.8	3.4:3.0∼3.8	3.7:3.3∼4.0	5.5:4.4∼6.3	<0.001* ^3^
iPTH (pg/mL) median: IQR	38:30∼47	36:30∼45	43:28∼59	88:67∼120	103:68∼165	<0.001* ^3^
Blood glucose (mg/dL) median: IQR	139:113∼179	142: 112∼180	131: 103∼165	135: 107∼197	-* ^6^	0.39* ^3^
Hb A1c (%) median: IQR	6.5:6.0∼7.0	6.6:6.1∼7.3	6.3:6.0∼6.9	6.6:5.7∼7.2	-* ^6^	0.33* ^3^
Use of active vitamin D n (%)	0 (0)	0 (0)	0 (0)	0 (0)	52 (51)	<0.001* ^4^

* 1: Stage 1 chronic kidney disease (CKD), estimated glomerular filtration rate (eGFR) ≥90 ml/min/1.73 m^2^; Stage 2 CKD, eGFR ≥60 to <90 ml/min/1.73 m^2^; Stage 3 CKD, eGFR ≥30 to <60 ml/min/1.73 m^2^; Stage 4 CKD, eGFR ≥15 to <30 ml/min/1.73 m^2^; and Stage 5 CKD, eGFR <15 ml/min/1.73 m^2^. *2: IQR: interquartile range. *3: P-value was evaluated with single ordered logistic regression model for eGFR stages. *4: P-value was calculated with chi-square test. *5: ACEI, angiotensin converting enzyme inhibitor; ARB, angiotensin II receptor blocker *6: The number of patients who could be measured was small and deleted from the analysis.

### Distribution of 25OHD and 1,25OHD

**Figure 1:**
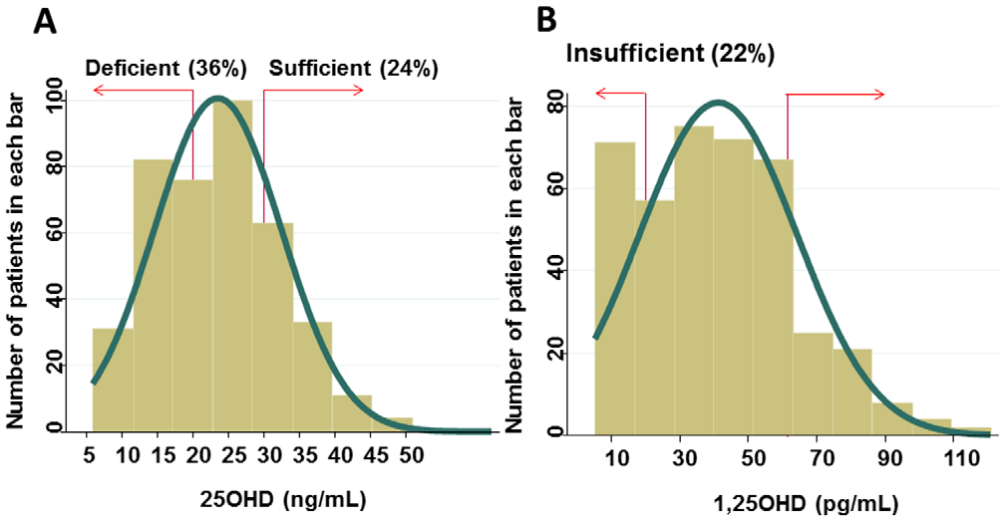
Histogram of circulating 25OHD levels (A) and 1, 25OHD levels (B) in patients with type 2 diabetes and with CKD stage 1∼5. Blood sampling was performed at entry; thus disease duration differed among patients. Serum 25OHD and 1,25OHD were measured by radioimmunoassay.

Histograms of circulating 25OHD (median [interquartile range: IQR], 23 [16∼29] ng/mL) and 1,25OHD levels (median [IQR], 40 [22∼57] pg/mL) are shown in Fig. 1. Almost one third and three quarters of patients showed deficient levels (<20 ng/mL) and insufficient (<30 ng/mL) levels of 25OHD, respectively [16] (Fig. 1A). On the other hand, 22% of patients demonstrated 1,25OHD levels below normal (Fig. 1B), and 94% of these patients had CKD stage 5.

### Annual oscillation in 25OHD and 1,25OHD levels

**Figure 2:**
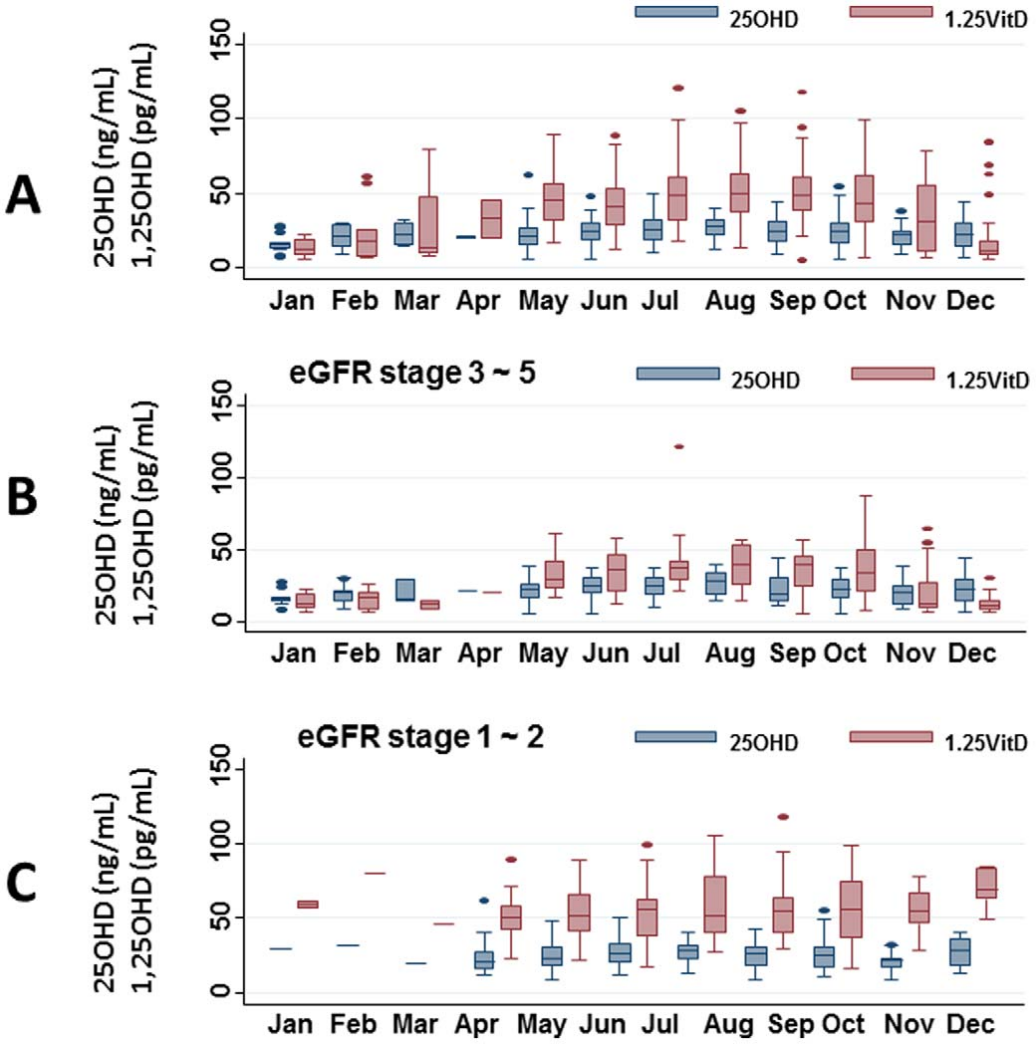
Serum 25OHD and 1,25OHD levels from January to December in patients with type 2 diabetes and with CKD stage 1∼5. Associations are shown using the total study population (A), a subpopulation with CKD stages 3∼5 (B), and a subpopulation with CKD stages 1∼2 (C). The central box extends from the 25^th^ to the 75^th^ percentile. All dots outside this range are outliers, which are not typical of the rest of the data.

There was an annual oscillation in 25OHD levels based on calendar month of blood sampling; levels were higher from June to October than in November (*P* = 0.001∼0.027) (Fig. 2A). In contrast, the annual oscillation in 1,25OHD levels was much more prominent than that for 25OHD levels. Compared with November, levels from May to October were significantly higher (P<0.001∼0.047), whereas levels from December to January were significantly lower (P<0.001∼ = 0.001). When we divided the study population into a group with CKD stage 3∼5 and a group with CKD stage 1∼2, the seasonal oscillation of 1,25OHD remained significant in the former group (Fig. 2B) but not significant in the latter group (Fig. 2C). 25OHD levels in CKD stage 1∼2 were significantly higher from July to October compared with November. On the other hand, 25OHD and 1,25OHD levels of CKD stage 1∼2 in December tended to be higher than in other months, but differences did not reach statistical significance.

### 25OHD/1,25OHD, and eGFR Levels

**Figure 3:**
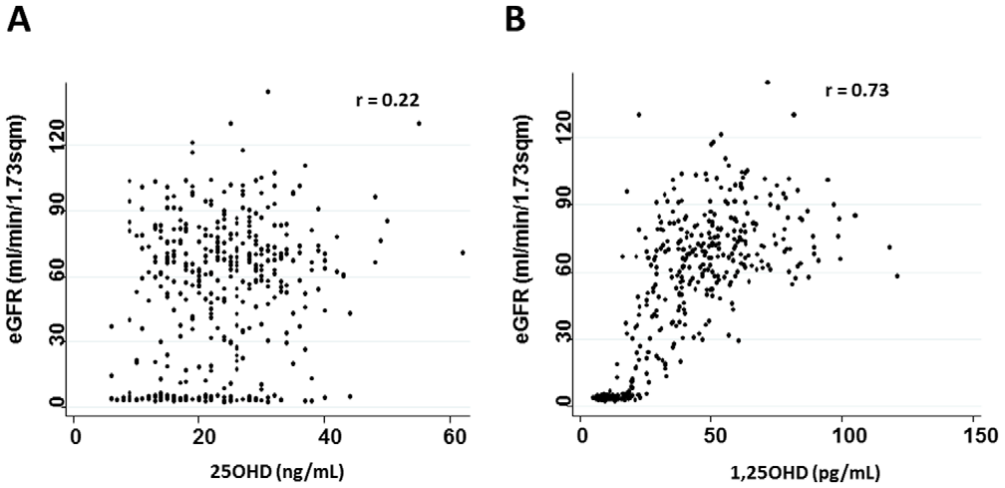
Two-way scatter graph for eGFR vs. 25OHD (A) or eGFR vs. 1,25OHD (B) in patients with type 2 diabetes and with CKD stage 1∼5.

eGFR levels were compared with 25OHD (Fig. 3A) and 1,25OHD (Fig. 3B) levels. eGFR levels were in direct proportion to both 25OHD (*P*<0.0001) and 1,25OHD levels (*P*<0.0001), but had a stronger linearity for 1,25OHD (r = 0.73) than 25OHD (r = 0.22). 1,25OHD levels were positively associated with in Ca levels (*P*<0.0001), whereas they were negatively associated with P levels (*P*<0.0001) and iPTH levels (*P*<0.0001).

### VDR Fok*I* polymorphisms, 25OHD/1,25OHD levels, and CKD stages

**Figure 4:**
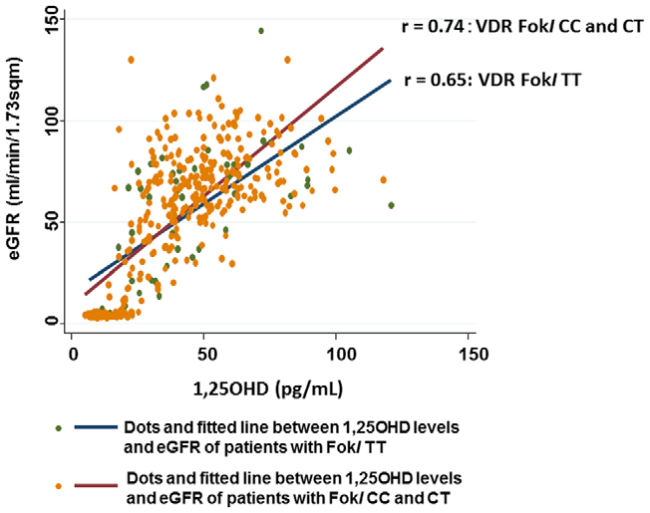
Two-way scatter graph for eGFR vs. 1,25OHD, stratified by patients with Fok*I* TT and with Fok*I* CC or CT. Fitting curves were drawn by calculating the prediction for eGFR from a linear regression of eGFR on 1,25OHD either in patients with Fok*I* TT and with Fok*I* CC or CT using STATA ver. 12.0.

First, Hardy-Weinberg equilibrium test did not show any significant findings for Fok*I* (P = 0.23): Genotype frequencies: CC: 175; CT: 177; TT: 58; Allele frequencies: C: 527; T: 293. Associations between VDR polymorphisms, serum 25OHD/1,25OHD levels, their interactions, and CKD stages were computed with a multiple ordered logistic regression model after adjustment for possible confounders including disease duration, calendar month, use of ACEI/ARB, use of statins, and levels of Ca, P, and iPTH (Table 2). Results showed that 1,25OHD levels (*P*<0.001), but not 25OHD levels, were negatively associated with CKD stage. Although Fok*I* polymorphisms by themselves showed no significant associations with CKD stages, a significant interaction between 1,25OHD and Fok*I*TT was observed (*P* = 0.008). The positive association between 1,25OHD and eGFR was steeper in patients with Fok*I*CC + CT polymorphisms (r = 0.74) than those with Fok*I*TT polymorphisms (r = 0.65) (Fig. 4). There was no interaction between 25OHD levels and Fok*I* polymorphisms.

**Table 2:** Multiple ordered logistic regression model to see the interaction between Fok*I* genotypes and 25OHD/1,25OHD levels on eGFR stage adjusted with 7 possible confounders*.

Variable	Odds ratio	95% Confidence interval	P value
25OHD	1.04	0.99 to 1.09	0.075
1,25OHD	0.94	0.92 to 0.96	<0.001
Fok*I* CC	Reference	-	-
Fok*I* CT	3.28	0.59 to 18.2	0.18
Fok*I* TT	0.17	0.02 to 1.44	0.10
25OHD in Fok*I* CC	Reference	-	-
25OHD in Fok*I* CT	0.97	0.91 to 1.03	0.33
25OHD in Fok*I* TT	0.98	0.90 to 1.06	0.57
1,25OHD in Fok*I* CC	Reference	-	-
1,25OHD in Fok*I* CT	1.00	0.97 to 1.02	0.83
1,25OHD in Fok*I* TT	1.05	1.01 to 1.08	0.008

* All variables in the table 2 as well as confounders disease duration; use of ACEI/ARB; use of statin; serum Ca, P, and iPTH levels; and calendar month were simultaneously computed with ordered logistic regression model.

## Discussion

In this study, we found that 1,25OHD levels, rather than 25OHD levels, showed seasonal oscillations in our study population. Peak values were seen from May to October and the lowest values were seen from December to February. Of interest, this oscillation of 1,25OHD levels was observed only in diabetic patients with advanced CKD (stage 3∼5), but not in patients with early CKD (stage 1∼2). Seasonal oscillations have been well documented in 25OHD levels [17], but to our knowledge, have not been reported in 1,25OHD levels, which is consistent with our previous study targeting patients with Parkinson's disease [14]. eGFR values were in direct proportion to both 25OHD and 1,25OHD levels, but they showed stronger linearity with 1,25OHD levels compared with 25OHD levels. A vicious cycle of 1,25OHD and renal dysfunction may make this association stronger than that of 25OHD levels. We hypothesized that 1OHase in the kidney was impaired by renal dysfunction, and when 25OHD levels decreased during winter, 1,25OHD levels also decreased in parallel to the levels of 25OHD.

In our study population of diabetic patients, Fok*I* polymorphisms by themselves showed no significant associations with CKD stage. A recent article showing the significance of Fok*I* polymorphisms compared patients with ESRD with healthy controls [18]. The study population was beta-thalassemia major, but patients with Fok*I*TT polymorphisms demonstrated impaired renal function with increased serum cystatin C levels, glomerular dysfunction with proteinuria, as well as significant tubulopathy with hypercalciuria and increased levels of urinary β_2_-microglobulin [19]. Moreover, a VDR haplotype between Bsm*I* and Taq*I* was protective against nephropathy in patients with type 1 diabetes, although Fok*I* polymorphisms had no significant association with the nephropathy [20]. In hypertensive patients, both higher 25OHD levels and Fok*I* T alleles were independently associated with lower plasma renin activity [21]. As described in the introduction, by switching from codon ATG (Fok*I*T) to ACG (Fok*I*C), they were considered to be more transcriptionally potent [11].

Our study showed a significant interaction between 1,25OHD and Fok*I*TT: The negative association between 1,25OHD and CKD stages was more repressed in Fok*I*TT polymorphisms than Fok*I*CC polymorphisms. Moreover, the positive association between 1,25OHD and eGFR was steeper in patients with Fok*I*CC + CT polymorphisms than those with Fok*I*TT polymorphisms. Some studies have reported interactions between VDR polymorphisms and 25OHD in diseases such as colorectal adenoma [22] and tuberculosis [23], although to our knowledge, this is the first time an interaction between VDR polymorphisms and 1,25OHD levels has been reported in diabetic patients with CKD.

There are several limitations to this study. First, the study design was cross-sectional. Thus, we cannot determine whether decreased 25OHD and 1,25OHD levels are an aggravate cause of CKD or a consequence of exacerbation of CKD in diabetic patients. There was a seasonal oscillation in 25OHD levels among different patients (i.e., not within the same patients). Thus information related to seasonal variations of 25OHD levels should be interpreted with caution. Second, interaction tests should be used cautiously in data analyses, as most studies are not powered to detect such interaction effects, and results of such tests are always exploratory in nature [24]. Third, we focused only on patients with type 2 diabetes. Low serum 25OHD levels were previously shown to be associated with an increased prevalence of type 2 diabetes as well as with insulin resistance [25]. However, our results may not be generalized to patients with CKD without diabetes.

In conclusion, these results suggest that higher 1,25OHD levels may be associated with better CKD stages in patients with type 2 diabetes and that this association was modified by Fok*I* polymorphisms.
